# Short-term safety and local tolerability of repeated exposure to aerosolized hypochlorous acid dry fog in healthy adults: a randomized controlled pilot study

**DOI:** 10.3389/fpubh.2026.1970564

**Published:** 2026-10-26

**Authors:** Tomasz Sanak, Marta Zakrzewska, Jacek Bil, Łukasz Krzowski, Grzegorz Witkowski, Zygmunt Mierczyk

**Affiliations:** 1 Jagiellonian University Medical College, Kraków, Poland; 2 MTZ Clinical Research MTZ Powered by Pratia, Warsaw, Poland; 3 Biodefense Laboratory, Biomedical Engineering Centre, Institute of Optoelectronics, Military University of Technology, Warsaw, Poland; 4 BioMedAqua Sp. z o.o., Dębica, Poland; 5 Biomedical Engineering Center, Institute of Optoelectronics, Military University of Technology, Warsaw, Poland

**Keywords:** aerosol exposure, dry fog, environmental disinfection, healthcare workers, hypochlorous acid, local tolerability, occupational health, safety

## Abstract

**Background:**

Hypochlorous acid (HOCI) is an oxidizing chlorine-based compound with broad antimicrobial activity and potential application as a dry-fog disinfectant in healthcare environments. However, human safety data for repeated exposure to aerosolized HOCI remain limited. This study evaluated the short-term safety and local tolerability of aerosolized HOCI dry fog under controlled conditions.

**Methods:**

This prospective, randomized, open-label pilot study included healthy adult volunteers aged 18–65 years. Participants were enrolled into two independent exposure cohorts and randomized 1:1 to aerosolized HOCI or aerosolized 0.9% sodium chloride. The investigational product was a 300-ppm HOCI solution dispersed as dry fog at 20 mL/m^3^. In the short repeated exposure cohort, participants underwent three 3-min exposures per day for 3 consecutive days. In the prolonged repeated exposure cohort, participants underwent one 60-min exposure per day for 9 consecutive days. Safety assessments included adverse events, vital signs, hematology, routine biochemistry, serum cytokines, laryngological examination with rhinoscopy, nasal cytology, and conjunctival assessment.

**Results:**

Eighty participants were randomized, 40 in each exposure cohort. No serious adverse events occurred, and no participant discontinued the study because of an adverse event. In the short repeated exposure cohort, adverse events were reported in 9/20 participants receiving HOCI and 3/20 receiving sodium chloride (45% vs. 15%; *p* = 0.082). In the prolonged repeated exposure cohort, adverse events were reported in 13/20 participants receiving HOCI and 0/20 receiving sodium chloride (65% vs. 0%; *p* < 0.001). All adverse events in the prolonged exposure cohort were mild; two were classified as definitely related to HOCI exposure. No persistent laryngological or conjunctival abnormalities were observed. Cytokine concentrations showed no consistent exposure-related pattern or sustained systemic inflammatory activation. Vital signs and routine laboratory parameters showed no clinically relevant systemic toxicity signal.

**Conclusion:**

Under the controlled exposure conditions tested, aerosolized HOCI dry fog was generally well tolerated in healthy adults. Prolonged repeated exposure was associated with more frequent non-serious, mild adverse events, but without serious adverse events, persistent local irritation, systemic inflammatory activation, or clinically meaningful laboratory toxicity.

## 1 Introduction

Environmental disinfection remains a fundamental component of infection prevention and control in healthcare settings (1, 2). Contaminated surfaces, reusable medical devices, and frequently touched equipment may contribute to the transmission of healthcare-associated pathogens (3, 4). Effective disinfection strategies should therefore combine broad antimicrobial activity with practical applicability, material compatibility, and an acceptable safety profile for patients and healthcare personnel (1, 5).

Hypochlorous acid (HOCI) is an oxidizing chlorine-based compound with broad antimicrobial activity and an established role in innate host defense. Previous studies have shown activity against bacteria (6, 7), viruses (8, 9), fungi, and spores (10). HOCI-based formulations have also been evaluated in fogging or atomization-based disinfection models, supporting their potential use for environmental decontamination beyond direct surface application (11, 12). Under appropriate conditions, HOCI decomposes into simple products and does not leave persistent toxic residue (13).

Dry-fog application creates a distinct exposure scenario. In contrast to manual surface disinfection, aerosolization disperses the disinfectant into the air and may therefore create potential respiratory, ocular, cutaneous, and mucosal exposure (12, 14). Moreover, the characteristics of gaseous or aerosolized HOCI may depend on environmental and exposure-related factors, including concentration, exposure duration, and decay over time (14).

Available human data from topical and wound-care applications of stabilized HOCI solutions suggest a favorable local tolerability profile (15, 16). However, these data cannot be directly extrapolated to repeated inhalational, ocular, or mucosal exposure during dry-fog disinfection. Experimental data suggest that the biological effects of aerosolized or gaseous HOCI may depend on concentration, exposure duration, and environmental conditions (17, 18). Therefore, controlled human exposure data are needed to assess whether repeated HOCI dry-fog exposure is associated with clinically relevant adverse events, local irritation, systemic inflammatory activation, or laboratory safety abnormalities.

The present prospective, randomized, open-label pilot study was designed to address this evidence gap by evaluating the short-term safety and local tolerability of repeated exposure to aerosolized HOCI dry fog compared with aerosolized 0.9% sodium chloride under controlled clinical conditions.

## 2 Materials and methods

### 2.1 Study design and oversight

This was a prospective, interventional, randomized, open-label pilot study designed to assess the safety and local tolerability of aerosolized HOCI used as a dry-fog disinfectant under controlled clinical conditions. The study was conducted at MTZ Clinical Research powered by Pratia, Warsaw, Poland, between May 5 and June 25, 2025.

The study was conducted in accordance with the Declaration of Helsinki, Good Clinical Practice principles and applicable regulatory requirements for medical experiments and biocidal products. The study protocol (HC/WA/09/24), informed consent form, and other required study documents were approved by the Bioethics Committee of the Warsaw Regional Medical Chamber, Warsaw, Poland (registration no. KB/1607/2025; resolution no. 8/25, March 20, 2025). Written informed consent was obtained from all participants. Study conduct and data quality were subject to independent clinical monitoring. Regular, external monitoring visits were performed during the study.

The study consisted of two independent cohorts that differed in protocol of exposure: a short repeated exposure cohort (Part A) and a prolonged repeated exposure cohort (Part B). Previous participation in Part A was an exclusion criterion for Part B.

### 2.2 Participants and analysis populations

Enrollment included consecutive healthy adult volunteers aged 18–65 years who were able to give written informed consent and follow the study schedule. At screening and enrolment, participants had to be free of active infection or communicable disease. Use of immunosuppressive drugs, corticosteroids, or other agents that could affect immune responses within the previous 30 days was not allowed. Both non-smokers and occasional smokers were eligible, however, occasional smokers (smoking no more than five cigarettes per day) must have agreed to cease smoking while at the study site.

Key exclusion criteria included a history of allergy or hypersensitivity to disinfectants or chemically related substances, including chlorine or HOCI; pregnancy or breastfeeding; chronic respiratory disease, including asthma or chronic obstructive pulmonary disease; active skin disease or other clinically relevant conditions that could affect study safety; participation in another clinical study within the preceding 30 days; psychiatric disorders potentially affecting consent or protocol compliance; claustrophobia or unwillingness to remain in a closed room; clinically significant abnormalities in screening laboratory tests; and employment by, or close relationship to employees of, the sponsor, manufacturer, or study site. The full analysis set included participants with at least one post-baseline assessment, and the per-protocol population included participants who completed the study without major protocol deviations.

In the short repeated exposure cohort, all randomized participants were incorporated into the safety, full analysis, and per-protocol populations. In the prolonged repeated exposure cohort, all randomized participants were included in the safety population. One participant randomized to HOCI withdrew on day 1 for personal reasons, unrelated to an adverse event; therefore, the per-protocol population in this cohort included 19 participants in the HOCI group and 20 participants in the sodium chloride group. There was no adverse event or serious adverse event leading to study discontinuation.

### 2.3 Randomization and interventions

Participants were randomized separately within each exposure cohort according to predefined randomization lists. The study was open-label, and treatment allocation was not masked. Within each cohort, participants were assigned 1:1 to aerosolized HOCI or aerosolized 0.9% sodium chloride.

The investigational product was BMAsept surf&air, an aqueous HOCI solution containing 300 ppm active chlorine, corresponding to 0.03%, with a pH of 4.5–5.5. The product was dispersed as a dry fog at a dose of 20 mL/m^3^. The comparator was aerosolized 0.9% sodium chloride administered under the same exposure conditions.

Aerosol was generated using a prototype dry-fog system developed by the Faculty of Mechanical Engineering of the Military University of Technology, Warsaw, Poland. The system consisted of a 3-kW compressor, a 50-L compressed-air reservoir, and automatic pressure control, and operated at 2 bar. The generated aerosol had a particle size of <9 μm, with a predominant fraction of approximately 5 μm. The mass median aerodynamic diameter was not directly measured.

Gaseous chlorine was measured indicatively after completion of aerosol generation, with no participants present in the exposure area, using a Dräger Pac 8000 Cl₂ portable detector. The detector was used for measurement of gaseous chlorine as Cl₂ only and did not quantify total HOCI-containing aerosol particles. The detector was equipped with an electrochemical Cl₂ sensor, catalog no. 6810890, serial no. 11RC1259, with a measurement range of 0–20 ppm, a detection limit of 0.05 ppm, and a resolution of 0.05 ppm. The last calibration before the study was performed on May 13, 2025.

#### 2.3.1 Short repeated exposure cohort

- *Exposure method and parameters*: Aerosol generation was performed using six aerosol-delivery nozzles positioned on the floor within the tent and directed upward at an angle of 45° according to the predefined setup (Supplementary Figure 1). During each cycle, 636 mL of solution was aerosolized, corresponding to 106 mL per nozzle and a nominal active chlorine dose of 6.0 mg/m^3^. The programmed aerosol-generation period was 5 min and 50 s, slightly longer than the theoretical delivery time, to ensure complete emptying of the buffer reservoirs. Initial room temperature and relative humidity before aerosol generation were 23 °C and 53%, respectively. The measured Cl₂ concentration after aerosol generation was 0.19 ppm, corresponding to approximately 0.55 mg/m^3^ using a conversion factor of 1 ppm Cl₂ ≈ 2.9 mg/m^3^.
- *Exposure environment*: Exposures were performed in a tent erected within a large hospital ward. The tent had an internal volume of 31.8 m^3^ and was fitted with a suspended white polyester inner lining with a surface density of 150–250 g/m^2^. The entrance consisted of overlapping fabric flaps secured with hook-and-loop fasteners to limit uncontrolled air leakage while allowing participant entry and exit. During aerosol generation and exposure, active ventilation inside the tent remained switched off.
- *Ventilation and aerosol removal*: After the 3-min exposure and after participants had left the tent, residual aerosol was extracted through ventilation ducts discharging outside both the tent and the hospital ward. Two inline duct fans, each with a nominal airflow capacity of 690 m^3^/h, were operated for 5 min. Based on the combined nominal airflow and internal tent volume, this corresponded to approximately 3.6 theoretical tent-volume equivalents.

#### 2.3.2 Prolonged repeated exposure cohort

- *Exposure method and parameters*: The same prototype aerosol-generation system was used, with floor-level nozzles positioned around the room according to the predefined layout (Supplementary Figure 2). During each cycle, 946 mL of solution was aerosolized, corresponding to a nominal active chlorine dose of 6.0 mg/m^3^. The aerosol-generation period was 8 min and 40 s. Initial room temperature and relative humidity before aerosol generation were 23 °C and 53%, respectively. The measured Cl₂ concentration after aerosol generation was 0.19 ppm, corresponding to approximately 0.55 mg/m^3^.
- *Exposure environment*: The 60-min exposure sessions were performed in a standard hospital room within the MTZ Clinical Research powered by Pratia early-phase clinical research unit. The room had an internal volume of 47.3 m^3^. During aerosol generation and the 60-min exposure period, active air exchange from the building ventilation system was suspended.
- *Ventilation and aerosol removal*: After participants left the room, building ventilation was reactivated. In addition, a single fan with a nominal airflow capacity of 690 m^3^/h was operated for 10 min through a flexible ventilation duct discharging outside the building to facilitate removal of residual aerosol. Consecutive exposure sessions were separated by approximately 24 h, during which the room remained subject to normal building ventilation.

### 2.4 Exposure regimens

In the *short repeated exposure cohort*, 40 participants were randomized, 20 to aerosolized HOCI and 20 to aerosolized 0.9% sodium chloride. Participants underwent repeated exposure over 3 consecutive days. Each day involved three separate 3-min exposure sessions, totaling 9 min of daily exposure and 27 min overall. Participants returned for follow-up assessments on days 5 and 10.

For the *extended repeated exposure group*, a distinct cohort of 40 participants was similarly randomized between aerosolized HOCI and aerosolized 0.9% sodium chloride (20 participants each). This group received a single 60-min exposure session daily over 9 consecutive days, accumulating 540 min of total exposure time. Their final follow-up evaluation occurred on day 20.

The two exposure regimens were selected to represent different anticipated exposure scenarios and to allow stepwise safety evaluation. The short repeated exposure cohort was intended to assess the safety of brief, repeated contacts with the aerosolized product, whereas the prolonged repeated exposure cohort was designed to evaluate a longer single daily exposure under controlled conditions. Progression to the prolonged repeated exposure cohort was allowed only after review of the safety profile of the short repeated exposure cohort by the principal investigator.

The prolonged repeated exposure cohort was initiated only after completion of the short repeated exposure cohort and review of its safety profile by the principal investigator.

### 2.5 Study assessments

Study assessments were performed according to prespecified visit schedules that differed between the two exposure cohorts. Screening was conducted within 28 days before the first exposure. The short repeated exposure cohort included three consecutive exposure days followed by safety follow-up visits on days 5 and 10, whereas the prolonged repeated exposure cohort included nine consecutive exposure days followed by a safety follow-up visit on day 20. Details of the prespecified visit schedule are provided in the Supplementary Methods and Supplementary Table 1.

At baseline, demographic data, medical history, concomitant medication use, eligibility criteria, physical examination findings, and randomization status were recorded. Physical examination included a general medical assessment, body weight, and height at screening. Serological screening was performed before randomization and included HIV 1/2, hepatitis B surface antigen, hepatitis B core antibody, and hepatitis C antibody testing. Urine pregnancy testing was performed in women of childbearing potential according to the study schedule.

Safety assessments were performed according to prespecified cohort-specific schedules and included adverse event monitoring, vital signs, hematology, routine biochemistry, cytokine measurements, and local tolerability assessments. Vital signs and blood samples were obtained at scheduled time points before and/or after exposure, depending on the exposure regimen, and were repeated during follow-up. Cytokine measurements included IL-1β, IL-6, IL-8, IL-10, IL-12, and TNF-α.

Local tolerability was assessed using (1) laryngological examination with rhinoscopy, (2) nasal cytology, and (3) conjunctival examination. Laryngological examination with rhinoscopy was used to evaluate the nasal mucosa, nasal septum, turbinates, pharynx, and laryngeal structures for potential irritant effects of exposure. Nasal swabs were obtained for cytological evaluation of epithelial morphology, goblet cells and inflammatory cells. Conjunctival assessment included evaluation of redness, edema, discharge, and other abnormalities, graded on a four-point scale: 0, no abnormality; 1, mild abnormality; 2, moderate abnormality; and 3, severe abnormality.

### 2.6 Endpoints

The primary endpoint was the safety of aerosolized HOCI exposure, assessed by the occurrence, frequency, severity, seriousness, outcome, and investigator-assessed relatedness of adverse events and serious adverse events throughout the study. Adverse events included any untoward medical occurrence reported by the participant or identified by the investigator, including respiratory symptoms, mucosal or ocular irritation, skin reactions, systemic symptoms, laboratory abnormalities, and other clinically relevant findings.

#### 2.6.1 Secondary endpoints

- changes from baseline in selected inflammatory and laboratory safety parameters,
- local tolerability findings,
- vital signs.

Inflammatory markers included serum concentrations of IL-1β, IL-6, IL-8, IL-10, IL-12, and TNF-α before and after exposure. Local tolerability findings included changes in laryngological examination with rhinoscopy, nasal cytology, and conjunctival assessment.

### 2.7 Safety monitoring

Safety was monitored continuously from the first exposure until study completion. Adverse events were assessed by the investigator for seriousness, severity, outcome, and causal relationship to the HOCI exposure and documented in the electronic case report form. Adverse events were coded using the Medical Dictionary for Regulatory Activities, version 28.0. Laboratory abnormalities and abnormal findings on local examinations were reported as adverse events when judged clinically significant by the investigator. Clinically relevant abnormalities and adverse events were followed until resolution, stabilization, or adequate medical explanation.

### 2.8 Statistical analysis

Because this was an exploratory pilot safety study, no formal sample size calculation was performed. The planned cohort size was 80 randomized participants, including 40 participants in the short repeated exposure (Part A) cohort and 40 participants in the prolonged repeated exposure cohort (Part B).

Continuous variables are presented as mean ± standard deviation (SD) or median with interquartile range (IQR), whereas categorical variables are expressed as absolute numbers and percentages. Between-group comparisons of continuous variables between the HOCI and NaCl groups at each study time point were performed using the independent-samples Welch’s *t*-test. Categorical variables were compared using Fisher’s exact test or the Fisher–Freeman–Halton exact test for contingency tables with more than two categories or small expected cell counts. All statistical tests were two-sided.

A *p-*value <0.05 was considered statistically significant. Owing to the exploratory nature of the study, no adjustment for multiple comparisons was applied; therefore, the results should be interpreted as hypothesis-generating.

## 3 Results

### 3.1 Study population and exposure

Overall, 80 consecutive healthy adult volunteers were randomized across two independent exposure cohorts. In the short repeated exposure cohort, 40 participants were randomized in a 1:1 ratio to aerosolized HOCI or aerosolized 0.9% sodium chloride. In the prolonged repeated exposure cohort, a separate group of 40 participants was similarly randomized in a 1:1 ratio.

Baseline demographic and anthropometric characteristics were comparable between the HOCI and sodium chloride groups within each exposure cohort (Table 1). All participants in the short repeated exposure cohort completed the study and were included in the safety, full analysis, and per-protocol populations. In the prolonged repeated exposure cohort, all randomized participants underwent at least one exposure and were included in the safety population. One participant assigned to HOCI withdrew on day 1 for personal reasons unrelated to an adverse event; therefore, the per-protocol population for this cohort included 19 participants in the HOCI group and 20 participants in the sodium chloride group. The detailed flowchart is presented in Figure 1.

**Table 1:** Baseline characteristics, participant disposition, and exposure regimens.

Variable	Short repeated exposure cohort (Part A)		*p*	Prolonged repeated exposure cohort (Part B)		*p*
	HOCI group (*n* = 20)	NaCl group (*n* = 20)		HOCI group (*n* = 19)	NaCl group (*n* = 20)	
Female sex, *n* (%)	13 (65)	12 (60)	0.74	9 (47)	9 (45)	1
Caucasian race, *n* (%)	20 (100)	20 (100)	1	19 (100)	20 (100)	1
Age, years	39.5 (34–48.5)	48.00 (35.00–50.25)	0.702	36.00 (29.75–41.25)	35.5 (29.5–47)	0.964
Height, cm	168.5 (162.5–175.5)	170.50 (167.75–179.00)	0.227	173 (164.5–178.25)	176.5 (168.75–181.5)	0.166
Body weight, kg	67.50 (64–81)	69.25 (60.50–88.25)	0.957	74 (60.75–95.5)	74.5 (69–85)	0.915
BMI, kg/m^2^	25.05 (21.58–26.95)	22.85 (20.03–27.58)	0.354	25.35 (22.58–29.52)	25 (23.55–26.2)	0.498
Exposure regimen	3 min×3/day×3 days	3 min × 3/day ×3 days	—	60 min/day × 9 days	60 min/day × 9 days	—
Total exposure time	27 min	27 min	—	540 min	540 min	—

Parts A and B were independent cohorts; previous participation in Part A was an exclusion criterion for Part B. Part A: short repeated exposure, 3 min × 3/day × 3 days.

Part B: prolonged repeated exposure, 60 min/day × 9 days.

BMI, body mass index; HOCI, hypochlorous acid; NaCl, sodium chloride.

**Figure 1:**
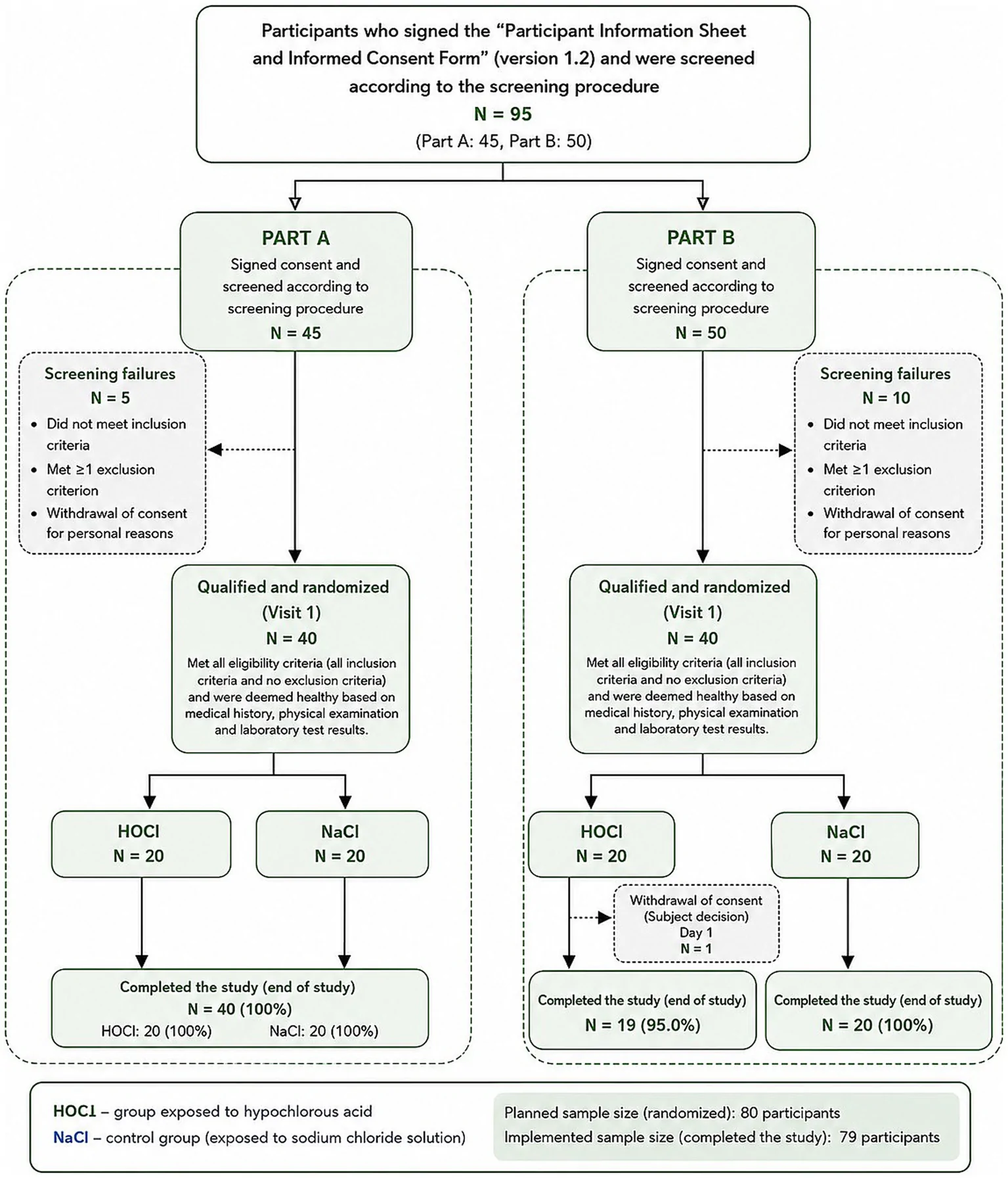
Flow of participants through the short repeated (Part A) and prolonged repeated (Part B) exposure cohorts.

### 3.2 Adverse events

Adverse event are summarized in Table 2 and depicted in Figure 2. The detailed adverse event terms provided in Supplementary Table 2. No serious adverse events occurred in either exposure cohort, and no participant discontinued the study because of an adverse event.

**Table 2:** Adverse events and safety outcomes by exposure regimen and treatment group.

Variable	Short repeated exposure cohort (Part A)		*p*	Prolonged repeated exposure cohort (Part B)		*p*
	HOCI group (*n* = 20)	NaCl group (*n* = 20)		HOCI group (*n* = 19) ^¥^	NaCl group (*n* = 20)	
Participants with ≥1 AE	9 (45%)	3 (15%)	0.08*	13 (65%)	0 (0%)	<0.001*
SAE	0 (0%)	0 (0%)	—	0 (0%)	0 (0%)	—
AE leading to study discontinuation	0 (0%)	0 (0%)	—	0 (0%)	0 (0%)	—
Severity distribution, events						
Mild AE	4 (20%)	3 (15%)	0.19	13 (65%)	0 (0%)	<0.001
Moderate AE	5 (25%)	0 (0%)		0 (0%)	0 (0%)	
Severe AE	0 (0%)	0 (0%)		0 (0%)	0 (0%)	
Causality distribution, events						
Unrelated AE	2 (10%)	1 (5%)	0.27	5 (25%)	0 (0%)	<0.001
Unlikely related AE	3 (15%)	0 (0%)		0 (0%)	0 (0%)	
Possibly related AE	4 (20%)	2 (10%)		6 (30%)	0 (0%)	
Definitely related AE	0 (0%)	0 (0%)		2 (10%)	0 (0%)	
Types of AE, events						
Neurological symptoms^‡^	5 (25%)	0 (0%)	0.056*	3 (15%)	0 (0%)	0.23*
Mucosal irritation^§^	1 (5%)	1 (5%)	0.46*	3 (15%)	0 (0%)	0.23*
Gastrointestinal symptoms^¶^	0 (0%)	0 (0%)	1*	3 (15%)	0 (0%)	0.23*
Hematological abnormalities^#^	2 (10%)	2 (10%)	1*	1 (5%)	0 (0%)	1*
Infectious events^**^	1 (5%)	0 (0%)	1*	2 (10%)	0 (0%)	0.47*

^¥^One participant withdrew consent on Day 1 without providing a reason. This was permitted by the protocol; no adverse event was documented as leading to discontinuation.

^*^Yates correction applied. ^‡^Headache, migraine, or dizziness. ^§^Throat irritation, conjunctival irritation, or rhinitis. ^¶^Nausea or vomiting. ^#^Leukopenia, neutropenia, or leukocytosis. ^**^Urinary tract infection, upper respiratory tract infection, or common cold.

*p*-values for severity and causality distributions are omnibus *p*-values calculated for the entire 3 × 2 or 4 × 2 distribution, respectively, and do not refer to individual rows. *p*-values for individual adverse-event categories were calculated as participant-level comparisons.

AE, adverse event; HOCI, hypochlorous acid; NaCl, sodium chloride; SAE, serious adverse event.

Parts A and B were independent cohorts; previous participation in Part A was an exclusion criterion for Part B. Part A: short repeated exposure, 3 min × 3/day × 3 days.

Part B: prolonged repeated exposure, 60 min/day × 9 days.

**Figure 2:**
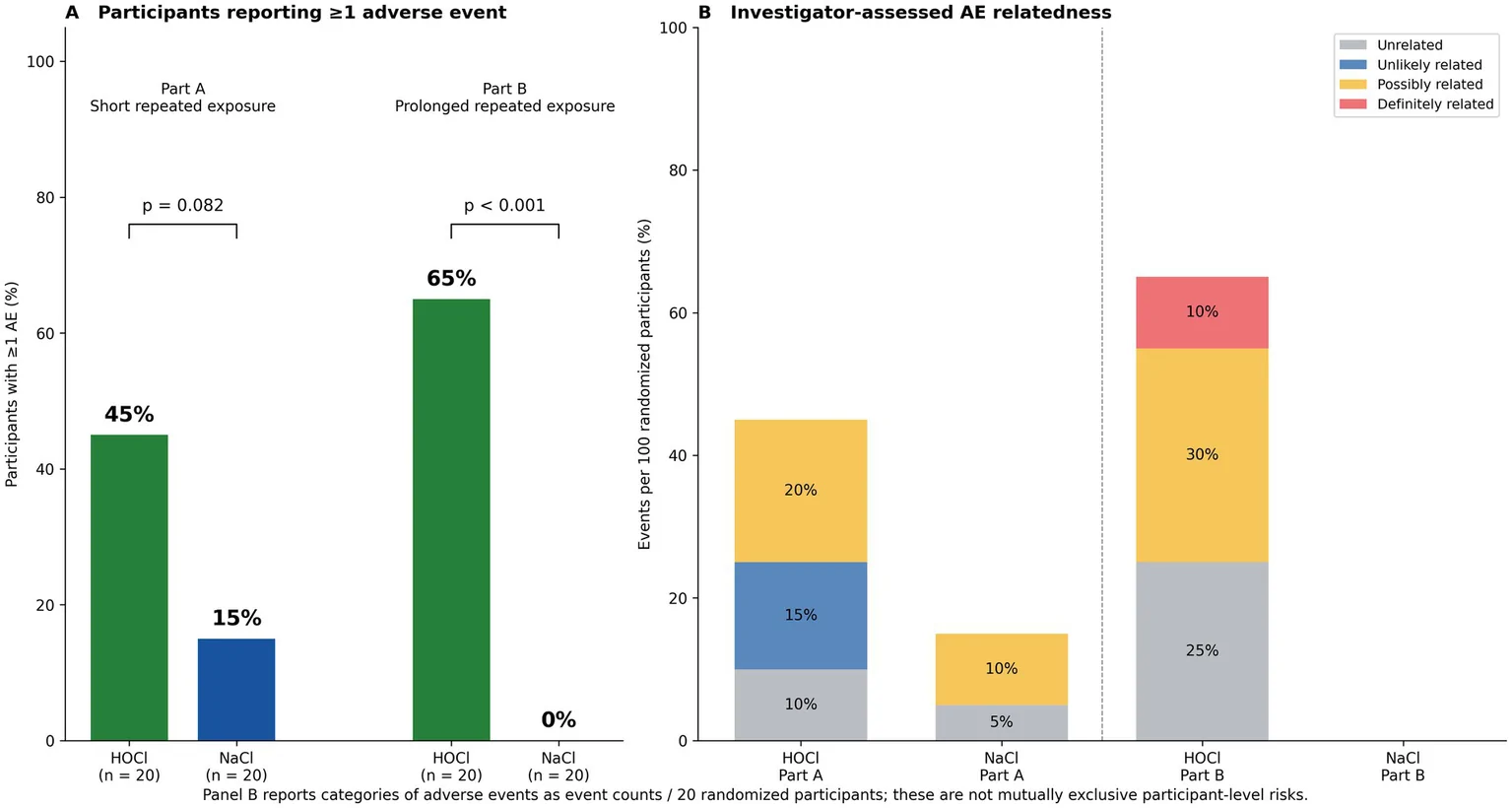
Adverse events according to exposure regimen and relationship to exposure. **(A)** Proportion of participants with at least one adverse event in the short repeated exposure cohort (Part A: three 3-min exposures per day for 3 consecutive days; total exposure time, 27 min) and the prolonged repeated exposure cohort (Part B: one 60-min exposure per day for 9 consecutive days; total exposure time, 540 min), according to assigned exposure: aerosolized HOCI or aerosolized 0.9% NaCl. **(B)** Investigator-assessed relationship of adverse events to the assigned exposure in Part A and Part B. AE, adverse event; NaCl, sodium chloride.

In the short repeated exposure cohort, adverse events were reported more frequently in the HOCI group than in the sodium chloride group, although this difference did not reach statistical significance. Events in this cohort were mild or moderate in severity, and no severe events were reported. The most frequent adverse events in the HOCI group were headache or migraine (5 events), followed by leukopenia or neutropenia (2 events), throat irritation (1 event), and urinary tract infection (1 event). In the sodium chloride group, single events of throat irritation, leukopenia, and neutropenia were reported. Hematological abnormalities occurred in both treatment groups, supporting a nonspecific pattern rather than a clear HOCI-related signal.

In the prolonged repeated exposure cohort, adverse events were significantly more frequent in the HOCI group than in the sodium chloride group. All reported events in this cohort were mild and resolved without serious clinical consequences. The most common grouped event categories were mucosal irritation, gastrointestinal symptoms, and neurological symptoms. Two events were classified as definitely related to HOCI exposure.

### 3.3 Inflammatory markers

Cytokine concentrations showed no consistent pattern suggesting clinically relevant systemic inflammatory activation after HOCI exposure (Table 3, Supplementary Tables 3A, 3B). The temporal profiles of cytokine concentrations are presented in Figure 3.

**Table 3:** Key inflammatory and local tolerability outcomes.

Variable	Short repeated exposure cohort (Part A)		*p*	Prolonged repeated exposure cohort (Part B)		*p*
	HOCI group (*n* = 20)	NaCl group (*n* = 20)		HOCI group (*n* = 19)*	NaCl group (*n* = 20)	
IL-1β baseline, pg./mL	2.46 ± 1.80	2.10 ± 1.21	0.458	2.59 ± 2.06	4.09 ± 2.53	0.049
IL-1β final exposure day, pg./mL	4.81 ± 2.76	3.65 ± 2.55	0.176	3.38 ± 2.47	2.98 ± 1.62	0.556
IL-1β change from baseline	+2.35 ± 2.21	+1.55 ± 2.06	0.240	+0.79 ± 2.06	−1.11 ± 2.03	0.006
IL-8 baseline, pg./mL	4.25 ± 2.37	4.76 ± 3.36	0.581	5.35 ± 3.66	5.91 ± 4.51	0.676
IL-8 peak during exposure, pg./mL	6.38 ± 8.30 (Day 3)	5.41 ± 3.49 (Day 2)	0.625	7.70 ± 5.68 (Day 4)	6.05 ± 3.61 (Day 1)	0.283
IL-8 final exposure day, pg./mL	6.38 ± 8.30	5.17 ± 2.04	0.479	4.42 ± 2.81	3.81 ± 1.09	0.374
IL-6 final exposure day, pg./mL	2.32 ± 0.61	2.45 ± 1.07	0.640	2.98 ± 0.97	3.15 ± 0.96	0.588
IL-10 final exposure day, pg./mL	2.35 ± 3.19	1.52 ± 0.28	0.270	1.52 ± 0.34	1.62 ± 0.31	0.347
TNF-α final exposure day, pg./mL	1.98 ± 0.84	2.07 ± 0.76	0.724	1.90 ± 0.74	2.01 ± 0.59	0.609
Clinically significant laryngological findings	0/20	0/20	—	0	0	—
Maximum post-exposure conjunctival abnormality	3(15%)	4(20%)	1.000†	1 (5.3%)	1 (5%)	1
Clinically relevant vital-sign change	0	0	-	0	0	—
Clinically relevant organ-toxicity signal in routine laboratory tests	0	0	—	0	0	—

Values for cytokines are mean ± SD. Final exposure day was Day 3 for Part A and Day 9 for Part B.

*One patient withdrew on day 1 for personal reasons.

HOCI, hypochlorous acid; IL, interleukin; NaCl, sodium chloride; TNF, tumor necrosis factor.

**Figure 3:**
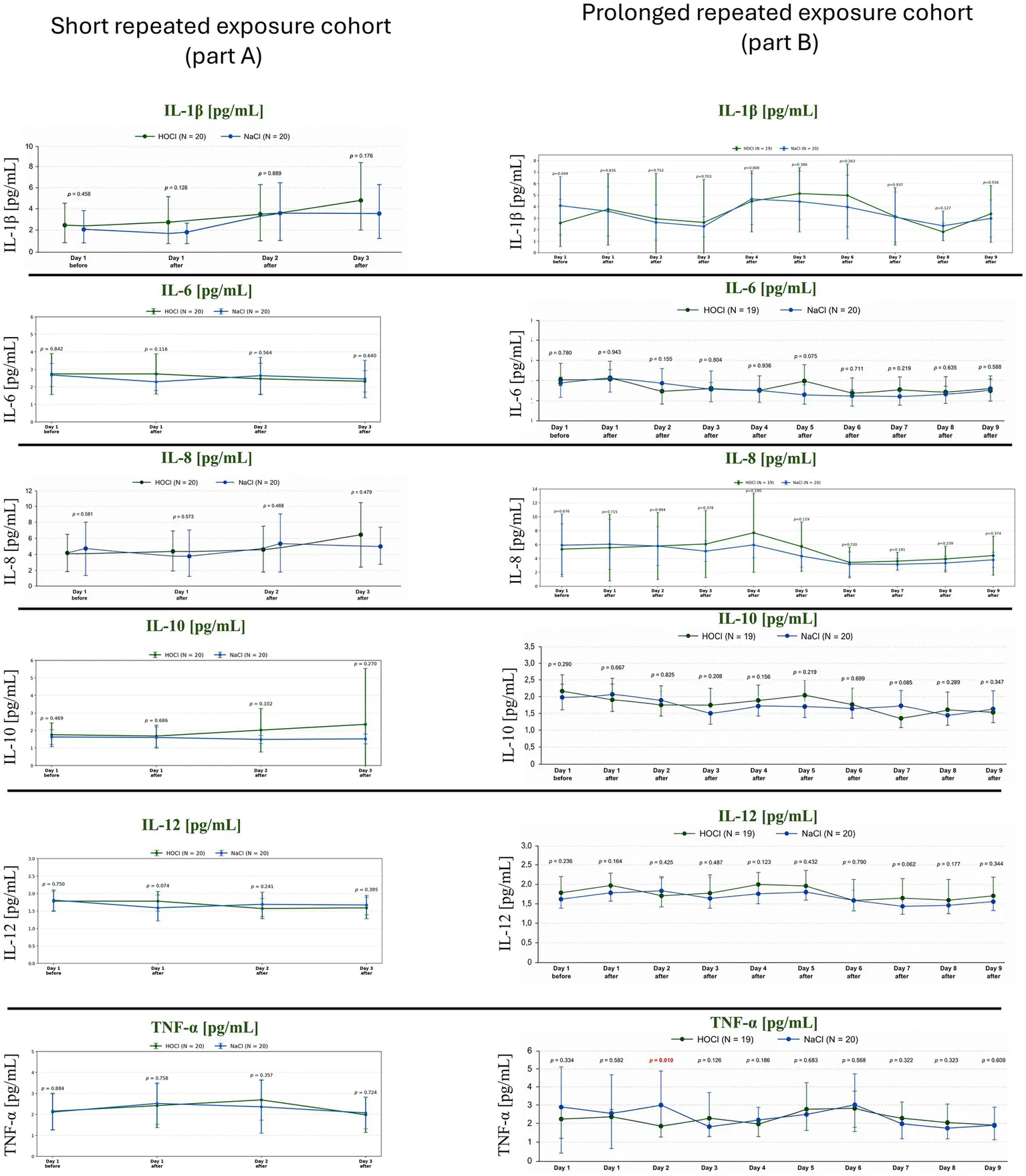
Serum cytokine concentrations according to exposure regimen and treatment group. Temporal changes in serum concentrations of IL-1β, IL-6, IL-8, IL-10, IL-12, and TNF-α in the short repeated exposure cohort (Part A: three 3-min exposures per day for 3 consecutive days; total exposure time, 27 min) and the prolonged repeated exposure cohort (Part B: one 60-min exposure per day for 9 consecutive days; total exposure time, 540 min), according to assigned exposure: aerosolized HOCI or aerosolized 0.9% NaCl. Values are shown for scheduled pre- and post-exposure time points. IL, interleukin; TNF, tumor necrosis factor.

In the short repeated exposure cohort, IL-1β and IL-8 showed modest numerical increases during exposure in both the HOCI and sodium chloride groups, with no statistically significant between-group differences on the final exposure day. IL-6, IL-10, IL-12, and TNF-α remained within a narrow range over time.

In the prolonged repeated exposure cohort, baseline IL-1β differed between groups, with higher values in the sodium chloride group. During repeated exposure, IL-1β and IL-8 fluctuated over time; however, final exposure-day values did not differ significantly between groups. Other cytokines, including IL-6, IL-10, IL-12, and TNF-α, did not show a consistent treatment-related pattern. Overall, the observed cytokine changes were transient and exploratory in nature.

### 3.4 Local tolerability

Local-tolerability heat maps are summarized in Table 3, Supplementary Figure 3 and Supplementary Table 4. In the short repeated exposure cohort, no clinically significant laryngological abnormalities were observed after exposure. Mild post-exposure conjunctival abnormalities were transient and occurred at a similar maximum frequency in the HOCI and sodium chloride groups: 15 and 20%, respectively; *p* = 1. No conjunctival abnormalities persisted at follow-up.

In the prolonged repeated exposure cohort, laryngological assessments were performed daily during the exposure period to evaluate potential local effects on the nasal and pharyngeal mucosa. Baseline laryngological findings were normal in the HOCI group and mostly normal in the sodium chloride group, with minor non-clinically significant abnormalities observed in 20% of sodium chloride participants; *p* = 0.11. Daily laryngological assessments showed that the mucosal appearance remained generally stable throughout the 9-day exposure period. In the HOCI group, transient mucosal redness was observed only sporadically, affecting 5.0% of participants after the first exposure day, 5.3% after the third exposure day, and 10.5% after the eighth exposure day. These findings were not accompanied by edema, discharge, pain, or need for treatment. In the sodium chloride group, isolated mild findings were also observed, including mucosal redness in 5.0% of participants after the seventh exposure day and minor septal mucosal injury in 5.0% after the eighth exposure day. Overall, no persistent local mucosal injury signal was identified.

Conjunctival abnormalities after exposure in the prolonged repeated exposure cohort were mild and transient. The maximum post-exposure frequency of mild conjunctival abnormalities was similar in the HOCI and sodium chloride groups: 5.3 and 5.0%, respectively; *p* = 1, with no persistent abnormalities at follow-up. Local tolerability findings by exposure regimen and treatment group are presented in Supplementary Figure 3.

### 3.5 Vital signs and routine laboratory safety

Vital signs remained stable throughout both exposure regimens (Supplementary Table 5). Isolated statistically significant between-group differences were observed at individual time points, but these were small in magnitude, inconsistent over time, and not considered clinically meaningful. No clinically relevant changes in blood pressure, heart rate, respiratory rate, or body temperature were observed.

Routine laboratory safety assessments did not reveal a clinically relevant organ-toxicity signal in either exposure cohort (Table 3). Hematological abnormalities were infrequent and occurred in both HOCI and sodium chloride groups, supporting their nonspecific character.

## 4 Discussion

In this prospective, randomized, open-label pilot study, aerosolized HOCI dry fog was generally well tolerated under controlled exposure conditions in healthy adults. No serious adverse events occurred, no participant discontinued the study because of an adverse event, and no persistent local irritation, clinically meaningful changes in vital signs, routine laboratory evidence of systemic toxicity, or consistent pattern of systemic inflammatory activation was observed. The main safety observation was a higher frequency of non-serious adverse events in the HOCI group during prolonged repeated exposure. These events were predominantly mild and mainly included transient mucosal irritation, gastrointestinal symptoms, and neurological symptoms.

The rationale for evaluating aerosolized HOCI is supported by previous studies demonstrating broad antimicrobial activity of HOCI-based agents. HOCI has been shown to act against bacteria, viruses, fungi, and spores, and has been evaluated in several applied disinfection settings (6, 9, 10, 19).

Previous preclinical work supported proceeding to controlled human exposure. *In vitro* dry-fog experiments using 300-ppm HOCI showed no significant cytotoxic or genotoxic effects under short-exposure conditions, and experiments including 500-ppm exposure supported tolerability under selected tested conditions (17). Before first human exposure, a Wistar rat safety study was conducted by the Jagiellonian University Medical College in Kraków, Poland, after ethics approval (authorization No. 665/2022). In this study, 238 animals were exposed to HOCI aerosol at 300 ppm and 20 mL/m^3^. No unequivocal indicators of acute toxicity, respiratory tract tissue injury or inflammation, or hepatic or renal injury were observed, irrespective of exposure duration or sequence. Low-concentration gaseous HOCI also did not impair respiratory epithelial cell viability or ciliary activity in laboratory models (18).

Dry-fog disinfection differs from surface application because aerosolization may expose the respiratory tract, ocular surface, skin, and mucous membranes. Although topical, wound-care, and nasal HOCI formulations have generally shown favorable local tolerability (15, 16, 20, 21), these observations cannot be directly extrapolated to repeated dry-fog exposure. Together with experimental data suggesting concentration-, duration-, and environment-dependent biological effects (17, 22). This provided the rationale for the present controlled human exposure study focused on upper-airway and conjunctival tolerability.

In the present study, adverse events were more frequent with HOCI than with sodium chloride during prolonged repeated dry-fog exposure, but all were mild, and none led to study discontinuation. Local assessments showed only sporadic, transient mucosal or conjunctival findings, without persistent injury or need for treatment. Nasal cytology did not indicate a consistent pattern of epithelial injury, cytotoxicity, or clinically relevant inflammatory response. These observations are compatible with prior topical and nasal HOCI data (15, 23), while extending the evidence to controlled aerosol exposure.

Systemic inflammatory and laboratory safety assessments provided further support for the absence of a clinically relevant systemic toxicity signal. Cytokine concentrations fluctuated over time, including numerical changes in IL-1β and IL-8, but these changes were inconsistent, exploratory, and did not form a coherent exposure-related pattern. Importantly, final exposure-day values did not suggest sustained systemic inflammatory activation. Prior experimental work indicates that HOCI-related biological effects are exposure-dependent and may vary according to concentration, duration, formulation, and experimental model. *In vitro* dry-fog exposure generated from a 300-ppm HOCI solution did not produce significant fibroblast injury under short-exposure conditions, whereas higher-concentration or longer repeated exposure was associated with cytotoxic and genotoxic effects (17). Conversely, low-concentration gaseous HOCI did not significantly affect cell viability or ciliary activity in human respiratory epithelial models (18). Our findings extend these experimental observations by providing controlled human exposure data and suggest no sustained systemic inflammatory or laboratory toxicity signal under the tested conditions.

The practical implication of these findings is that aerosolized HOCI dry fog, at the tested concentration and under controlled exposure conditions, showed a reassuring short-term safety and local tolerability profile in healthy adults. However, the results should not be interpreted as evidence of unrestricted safety across all populations or real-world exposure scenarios. Healthcare environments are heterogeneous, and exposure may vary according to room size, ventilation, fogging device, concentration, duration, frequency of use, and timing of human re-entry. In addition, patients with asthma, chronic obstructive pulmonary disease, cardiovascular disease, chronic inflammatory conditions, immunosuppression, advanced age, or increased mucosal sensitivity may respond differently from the healthy adults enrolled in this study.

## 5 Strengths and limitations

This study has several strengths. It was a prospective, randomized, controlled pilot study conducted under predefined and monitored exposure conditions, with aerosolized 0.9% sodium chloride used as an active comparator for dry-fog exposure. The assessment included two independent exposure regimens, representing short repeated and prolonged repeated exposure, and incorporated a broad safety evaluation, including adverse events, vital signs, routine laboratory tests, inflammatory markers, laryngological examination, nasal cytology, and conjunctival assessment. Study conduct was supported by clinical monitoring and source-data verification, strengthening the reliability of the collected safety data.

The main limitations are the exploratory design, small sample size, and lack of formal power calculation, which preclude reliable detection of rare adverse events or small between-group differences. The open-label design may have influenced reporting or assessment of mild subjective symptoms. The inclusion of healthy adults aged 18–65 years limits generalizability to children, older adults, patients with chronic respiratory or cardiovascular disease, and other vulnerable populations. In addition, exposure was performed under controlled clinical conditions, with follow-up limited to 10 days after short repeated exposure and 20 days after prolonged repeated exposure; therefore, delayed, cumulative, or real-world effects in healthcare settings require further exploration.

## 6 Conclusion

Under the controlled exposure conditions tested, aerosolized HOCI was generally well tolerated. The main safety signal was a higher frequency of non-serious, predominantly mild adverse events in the prolonged repeated exposure cohort. No serious adverse events, adverse event-related discontinuations, persistent local irritation, clinically meaningful changes in vital signs, routine laboratory evidence of systemic toxicity, or consistent pattern of clinically relevant systemic inflammatory activation were observed.

## Data Availability

The raw data supporting the conclusions of this article will be made available by the authors, without undue reservation.
