# Supplementary material for: Short-term safety and local tolerability of repeated exposure to aerosolized hypochlorous acid dry fog in healthy adults: a randomized controlled pilot study
Source: Front Public Health. 2026 Oct 26;14:1970564. doi: 10.3389/fpubh.2026.1970564 (PMC13746904; doi:10.3389/fpubh.2026.1970564)
Supplement: Supplementary file 1 [file Data_Sheet_1.docx]

Supplementary Methods

Schedule of study assessments

In the **short repeated exposure cohort**, participants attended five study visits after screening: exposure visits on days 1, 2, and 3, and follow-up visits on days 5 and 10. On each exposure day, participants underwent three 3-minute exposure cycles separated by 3-hour intervals. Vital signs, hematology, routine biochemistry, cytokines, laryngological examination, nasal cytology, conjunctival assessment, concomitant medication review, and adverse-event assessment were performed according to the prespecified schedule. Post-exposure vital-sign measurements and blood sampling were performed after leaving the exposure room, with a target interval of at least 10 minutes. Conjunctival assessment was performed before exposure and after each exposure cycle on days 1–3, and again at follow-up visits.

In the **prolonged repeated exposure cohort**, participants attended ten study visits after screening: exposure visits on days 1–9 and a follow-up visit on day 20. On each exposure day, participants underwent one 60-minute exposure cycle. Vital signs, hematology, routine biochemistry, cytokines, laryngological examination, nasal cytology, conjunctival assessment, concomitant medication review, and adverse-event assessment were performed according to the prespecified schedule. Post-exposure vital-sign measurements and blood sampling were performed after leaving the exposure room, with a target interval of at least 10 minutes. Conjunctival assessment was performed before exposure on day 1, after exposure on days 1–9, and at the day-20 follow-up visit.

Serological screening was performed during screening only. Urine pregnancy testing was performed in women of childbearing potential at screening, before exposure on day 1, and at the final follow-up visit.

Schedule of study assessments (Supplementary Methods)

| **Assessment** | **Short repeated exposure cohort** | **Prolonged repeated exposure cohort** |
| --- | --- | --- |
| Screening | Within 28 days before first exposure | Within 28 days before first exposure |
| Number of post-screening visits | 5 visits: days 1, 2, 3, 5, and 10 | 10 visits: days 1–9 and day 20 |
| Exposure visits | Days 1–3 | Days 1–9 |
| Follow-up visits | Days 5 and 10 | Day 20 |
| Exposure schedule | Three 3-minute exposures per day, separated by 3-hour intervals, for 3 days | One 60-minute exposure per day for 9 days |
| Total exposure time | 27 minutes | 540 minutes |
| Demographics and medical history | Screening/day 1 | Screening/day 1 |
| Physical examination | Screening/day 1 and final follow-up | Screening/day 1 |
| Vital signs | Before exposure on day 1, after final exposure on days 1–3, and day 10 | Before exposure on day 1, after exposure on days 1–9, and day 20 |
| Hematology and routine biochemistry | Screening, before exposure on day 1, after final exposure on days 1–3, and day 10 | Screening, before exposure on day 1, after exposure on days 1–9, and day 20 |
| Cytokines | Before exposure on day 1 and after final exposure on days 1–3 | Before exposure on day 1 and after exposure on days 1–9 |
| Laryngological examination with rhinoscopy | Before exposure on day 1 and after final exposure on days 1–3 | Before exposure on day 1 and after exposure on days 1–9 |
| Nasal cytology | Before exposure on day 1 and after final exposure on days 1–3 | Before exposure on day 1 and after exposure on days 1–9 |
| Conjunctival assessment | Before exposure and after each exposure cycle on days 1–3; days 5 and 10 | Before exposure on day 1, after exposure on days 1–9, and day 20 |
| Serology | Screening only | Screening only |
| Urine pregnancy test | Screening, day 1, and day 10 | Screening, day 1, and day 20 |
| Concomitant medication review | Each study visit | Each study visit |
| AE/SAE monitoring | Each study visit from first exposure to final follow-up | Each study visit from first exposure to final follow-up |

Supplementary Table 1. Detailed adverse events and their incidence.

|  | Short repeated exposure cohort (part A) | | P | Prolonged repeated exposure cohort (part B) | | P |
| --- | --- | --- | --- | --- | --- | --- |
| Variable | HOCl group  (n=20) | NaCl group  (n=20) |  | HOCl group (n=20; safety population) | NaCl group  (n=20) |  |
| Participants with >=1 AE | 9 (45%) | 3 (15%) | 0.082 | 13 (65%) | 0 (0%) | <0.001 |
| Headache | 3 | 0 | 0.231 | 2 | 0 | 0.487 |
| Migraine | 2 | 0 | 0.487 | 0 | 0 | - |
| Dizziness | 0 | 0 | - | 1 | 0 | 1 |
| Throat irritation | 1 | 1 | 1 | 1 | 0 | 1 |
| Conjunctival irritation | 0 | 0 | - | 1 | 0 | 1 |
| Rhinitis | 0 | 0 | - | 1 | 0 | 1 |
| Nausea | 0 | 0 | - | 2 | 0 | 0.49 |
| Vomiting | 0 | 0 | - | 1 | 0 | 1 |
| Leukopenia | 1 | 1 | 1 | 0 | 0 | - |
| Neutropenia | 1 | 1 | 1 | 0 | 0 | - |
| Leukocytosis | 0 | 0 | - | 1 | 0 | 1 |
| Urinary tract infection | 1 | 0 | - | 0 | 0 | - |
| Upper respiratory tract infection | 0 | 0 | - | 1 | 0 | 1 |
| Common cold | 0 | 0 | - | 1 | 0 | 1 |
| Weakness | 0 | 0 | - | 1 | 0 | 1 |
| SAE | 0 | 0 | - | 0 | 0 | - |

The first row reports the number and proportion of participants with at least one adverse event in the safety population. Other rows reproduce the reported counts of individual adverse-event terms; these counts should not be summed to estimate the number of affected participants, because multiple terms may occur in one participant. The denominator for the Part B HOCl safety population is N = 20; 19 participants completed the protocol.

Abbreviations: AE, adverse event; HOCl, hypochlorous acid; NaCl, sodium chloride; SAE, serious adverse event.

Supplementary Table 2. Cytokine concentrations in the short repeated exposure cohort.

| Cytokine | Time point | HOCl group  (n=20) | NaCl group  (n=20) | P |
| --- | --- | --- | --- | --- |
| IL-1β | Day 1 before exposure | 2.46 ± 1.80; 1.85 (1.45–2.48) | 2.10 ± 1.21; 1.65 (1.48–2.10) | 0.458 |
| IL-1β | Day 1 after exposure | 2.79 ± 2.39; 1.75 (1.38–2.88) | 1.83 ± 0.95; 1.65 (1.30–1.80) | 0.128 |
| IL-1β | Day 2 after exposure | 3.55 ± 2.62; 2.10 (1.70–5.40) | 3.67 ± 2.73; 2.45 (1.88–4.15) | 0.889 |
| IL-1β | Day 3 after exposure | 4.81 ± 2.76; 7.20 (1.58–7.20) | 3.65 ± 2.55; 2.20 (1.58–7.20) | 0.176 |
| IL-6 | Day 1 before exposure | 2.74 ± 1.17; 2.30 (2.10–2.50) | 2.68 ± 0.66; 2.80 (2.30–3.03) | 0.842 |
| IL-6 | Day 1 after exposure | 2.74 ± 1.15; 2.35 (1.90–3.05) | 2.29 ± 0.49; 2.20 (1.88–2.50) | 0.116 |
| IL-6 | Day 2 after exposure | 2.46 ± 0.90; 2.25 (2.08–2.53) | 2.64 ± 1.05; 2.45 (2.22–2.65) | 0.564 |
| IL-6 | Day 3 after exposure | 2.32 ± 0.61; 2.20 (1.98–2.53) | 2.45 ± 1.07; 2.10 (1.80–2.78) | 0.640 |
| IL-8 | Day 1 before exposure | 4.25 ± 2.37; 3.55 (2.95–4.40) | 4.76 ± 3.36; 4.00 (3.18–5.23) | 0.581 |
| IL-8 | Day 1 after exposure | 4.55 ± 2.34; 4.15 (3.20–4.68) | 4.11 ± 2.54; 3.50 (2.55–4.20) | 0.573 |
| IL-8 | Day 2 after exposure | 4.67 ± 2.83; 3.85 (3.65–4.78) | 5.41 ± 3.49; 4.15 (3.70–5.00) | 0.468 |
| IL-8 | Day 3 after exposure | 6.38 ± 8.30; 4.20 (3.78–4.85) | 5.17 ± 2.04; 4.35 (4.08–5.48) | 0.479 |
| IL-10 | Day 1 before exposure | 1.76 ± 0.68; 1.55 (1.40–1.73) | 1.63 ± 0.42; 1.50 (1.40–1.60) | 0.469 |
| IL-10 | Day 1 after exposure | 1.68 ± 0.63; 1.50 (1.28–1.65) | 1.60 ± 0.61; 1.50 (1.30–1.63) | 0.686 |
| IL-10 | Day 2 after exposure | 2.02 ± 1.24; 1.60 (1.40–1.85) | 1.49 ± 0.23; 1.50 (1.30–1.60) | 0.102 |
| IL-10 | Day 3 after exposure | 2.35 ± 3.19; 1.50 (1.30–1.90) | 1.52 ± 0.28; 1.50 (1.38–1.73) | 0.270 |
| IL-12 | Day 1 before exposure | 1.78 ± 0.29; 1.90 (1.80–1.90) | 1.81 ± 0.30; 1.90 (1.68–1.90) | 0.750 |
| IL-12 | Day 1 after exposure | 1.78 ± 0.28; 1.85 (1.68–1.90) | 1.59 ± 0.37; 1.75 (1.30–1.90) | 0.074 |
| IL-12 | Day 2 after exposure | 1.57 ± 0.29; 1.55 (1.30–1.90) | 1.69 ± 0.35; 1.70 (1.48–1.90) | 0.241 |
| IL-12 | Day 3 after exposure | 1.59 ± 0.31; 1.65 (1.38–1.90) | 1.67 ± 0.28; 1.80 (1.40–1.90) | 0.395 |
| TNF-α | Day 1 before exposure | 2.15 ± 0.87; 2.00 (1.48–2.63) | 2.11 ± 0.86; 1.80 (1.58–2.53) | 0.884 |
| TNF-α | Day 1 after exposure | 2.42 ± 1.05; 2.35 (1.48–3.70) | 2.52 ± 0.99; 1.95 (1.70–3.70) | 0.758 |
| TNF-α | Day 2 after exposure | 2.69 ± 0.96; 2.45 (1.70–3.70) | 2.36 ± 1.26; 1.80 (1.48–2.95) | 0.357 |
| TNF-α | Day 3 after exposure | 1.98 ± 0.84; 1.60 (1.40–2.10) | 2.07 ± 0.76; 1.85 (1.55–2.30) | 0.724 |

Values are mean ± SD; median (IQR). P values compare HOCl with NaCl at the corresponding time point in the source report.

Abbreviations: HOCl, hypochlorous acid; IL, interleukin; NaCl, sodium chloride; TNF, tumor necrosis factor.

Supplementary Table 3. Cytokine concentrations in the prolonged repeated exposure cohort.

| Cytokine | Time point | HOCl group*  (n=19) | NaCl group  (n=20) | P |
| --- | --- | --- | --- | --- |
| IL-1β | Day 1 before exposure | 2.59 ± 2.06; 2.10 (1.55–2.85) | 4.09 ± 2.53; 3.10 (2.10–6.13) | 0.049 |
| IL-1β | Day 1 after exposure | 3.78 ± 3.11; 2.15 (1.90–7.20) | 3.60 ± 2.14; 2.65 (2.08–4.70) | 0.835 |
| IL-1β | Day 2 after exposure | 2.95 ± 3.96; 1.80 (1.60–2.05) | 2.64 ± 1.53; 2.10 (1.60–3.20) | 0.752 |
| IL-1β | Day 3 after exposure | 2.63 ± 3.74; 1.80 (1.55–2.00) | 2.29 ± 0.88; 2.05 (1.78–2.55) | 0.703 |
| IL-1β | Day 4 after exposure | 4.47 ± 2.65; 3.70 (2.05–7.20) | 4.67 ± 2.21; 4.00 (2.73–7.20) | 0.800 |
| IL-1β | Day 5 after exposure | 5.14 ± 2.26; 4.70 (3.60–7.20) | 4.45 ± 2.64; 4.55 (1.68–7.20) | 0.386 |
| IL-1β | Day 6 after exposure | 4.98 ± 2.72; 7.20 (1.75–7.20) | 3.98 ± 2.77; 2.30 (1.45–7.20) | 0.263 |
| IL-1β | Day 7 after exposure | 3.16 ± 2.49; 1.70 (1.45–5.20) | 3.10 ± 2.18; 1.95 (1.70–3.80) | 0.937 |
| IL-1β | Day 8 after exposure | 1.81 ± 0.77; 1.60 (1.40–1.80) | 2.34 ± 1.29; 1.90 (1.60–2.45) | 0.127 |
| IL-1β | Day 9 after exposure | 3.38 ± 2.47; 2.00 (1.55–6.20) | 2.98 ± 1.62; 2.35 (1.90–3.35) | 0.556 |
| IL-6 | Day 1 before exposure | 4.03 ± 2.54; 3.70 (2.88–4.15) | 3.85 ± 1.22; 3.50 (2.88–4.65) | 0.780 |
| IL-6 | Day 1 after exposure | 4.09 ± 1.99; 3.50 (2.90–4.80) | 4.13 ± 1.42; 3.80 (3.23–4.97) | 0.943 |
| IL-6 | Day 2 after exposure | 2.88 ± 0.73; 2.80 (2.40–3.40) | 3.61 ± 2.04; 3.05 (2.20–4.13) | 0.155 |
| IL-6 | Day 3 after exposure | 3.04 ± 0.67; 2.80 (2.55–3.60) | 3.11 ± 1.09; 2.85 (2.30–3.65) | 0.804 |
| IL-6 | Day 4 after exposure | 3.01 ± 0.91; 2.80 (2.40–3.50) | 2.98 ± 1.24; 2.60 (2.48–3.30) | 0.936 |
| IL-6 | Day 5 after exposure | 3.82 ± 2.78; 3.00 (2.40–4.00) | 2.60 ± 1.03; 2.45 (2.00–3.00) | 0.075 |
| IL-6 | Day 6 after exposure | 2.78 ± 2.47; 2.20 (1.95–2.35) | 2.54 ± 1.49; 2.20 (1.60–2.58) | 0.711 |
| IL-6 | Day 7 after exposure | 2.95 ± 1.39; 2.30 (2.10–3.25) | 2.50 ± 0.72; 2.50 (1.80–3.10) | 0.219 |
| IL-6 | Day 8 after exposure | 2.71 ± 0.82; 2.60 (2.15–3.35) | 2.84 ± 0.87; 2.60 (2.10–3.40) | 0.635 |
| IL-6 | Day 9 after exposure | 2.98 ± 0.97; 2.90 (2.55–3.40) | 3.15 ± 0.96; 3.05 (2.45–3.68) | 0.588 |
| IL-8 | Day 1 before exposure | 5.35 ± 3.66; 4.30 (3.80–5.20) | 5.91 ± 4.51; 4.45 (3.80–5.33) | 0.676 |
| IL-8 | Day 1 after exposure | 5.55 ± 4.80; 3.95 (3.50–4.73) | 6.05 ± 3.61; 5.45 (3.68–7.08) | 0.715 |
| IL-8 | Day 2 after exposure | 5.80 ± 4.82; 4.50 (3.70–5.20) | 5.79 ± 2.78; 4.80 (3.98–7.15) | 0.994 |
| IL-8 | Day 3 after exposure | 6.08 ± 4.83; 4.60 (4.15–5.60) | 5.05 ± 1.47; 4.60 (4.08–5.40) | 0.378 |
| IL-8 | Day 4 after exposure | 7.70 ± 5.68; 6.00 (5.00–6.75) | 5.96 ± 1.84; 5.55 (4.70–6.83) | 0.190 |
| IL-8 | Day 5 after exposure | 5.71 ± 3.57; 5.20 (3.65–6.35) | 4.34 ± 1.58; 4.15 (3.18–5.05) | 0.119 |
| IL-8 | Day 6 after exposure | 3.43 ± 2.21; 2.70 (2.45–3.40) | 3.20 ± 1.76; 3.05 (2.18–3.45) | 0.720 |
| IL-8 | Day 7 after exposure | 3.62 ± 1.28; 3.30 (2.75–4.05) | 3.16 ± 0.85; 3.05 (2.80–3.30) | 0.191 |
| IL-8 | Day 8 after exposure | 3.93 ± 1.87; 3.20 (2.95–3.90) | 3.35 ± 1.01; 3.05 (2.88–3.35) | 0.239 |
| IL-8 | Day 9 after exposure | 4.42 ± 2.81; 3.90 (2.85–4.35) | 3.81 ± 1.09; 4.00 (2.90–4.50) | 0.374 |
| IL-10 | Day 1 before exposure | 2.16 ± 0.49; 2.10 (1.90–2.33) | 2.01 ± 0.37; 1.90 (1.78–2.23) | 0.290 |
| IL-10 | Day 1 after exposure | 1.96 ± 0.43; 1.80 (1.68–2.15) | 2.04 ± 0.68; 1.95 (1.68–2.30) | 0.669 |
| IL-10 | Day 2 after exposure | 1.77 ± 0.75; 1.50 (1.20–1.85) | 1.83 ± 0.87; 1.50 (1.38–1.83) | 0.825 |
| IL-10 | Day 3 after exposure | 1.76 ± 0.71; 1.40 (1.35–1.80) | 1.51 ± 0.48; 1.40 (1.28–1.60) | 0.208 |
| IL-10 | Day 4 after exposure | 1.89 ± 0.36; 1.90 (1.70–1.95) | 1.75 ± 0.22; 1.70 (1.58–1.90) | 0.156 |
| IL-10 | Day 5 after exposure | 2.02 ± 0.78; 2.00 (1.50–2.20) | 1.73 ± 0.64; 1.60 (1.20–1.85) | 0.219 |
| IL-10 | Day 6 after exposure | 1.78 ± 0.81; 1.40 (1.20–1.75) | 1.68 ± 0.84; 1.30 (1.10–1.55) | 0.699 |
| IL-10 | Day 7 after exposure | 1.40 ± 0.26; 1.30 (1.20–1.55) | 1.71 ± 0.69; 1.50 (1.30–1.60*) | 0.085 |
| IL-10 | Day 8 after exposure | 1.63 ± 0.54; 1.50 (1.35–1.70) | 1.46 ± 0.45; 1.30 (1.30–1.40) | 0.289 |
| IL-10 | Day 9 after exposure | 1.52 ± 0.34; 1.50 (1.30–1.60) | 1.62 ± 0.31; 1.50 (1.48–1.73) | 0.347 |
| IL-12 | Day 1 before exposure | 1.81 ± 0.49; 1.75 (1.50–2.00) | 1.65 ± 0.33; 1.75 (1.38–1.90) | 0.236 |
| IL-12 | Day 1 after exposure | 1.99 ± 0.50; 1.90 (1.78–2.20) | 1.80 ± 0.31; 1.90 (1.63–1.90) | 0.164 |
| IL-12 | Day 2 after exposure | 1.70 ± 0.47; 1.70 (1.35–1.90) | 1.85 ± 0.69; 1.90 (1.58–1.90) | 0.425 |
| IL-12 | Day 3 after exposure | 1.79 ± 0.67; 1.70 (1.30–1.90) | 1.67 ± 0.35; 1.65 (1.45–1.90) | 0.487 |
| IL-12 | Day 4 after exposure | 2.02 ± 0.59; 1.90 (1.65–2.20) | 1.78 ± 0.34; 1.80 (1.58–2.03) | 0.123 |
| IL-12 | Day 5 after exposure | 1.98 ± 0.82; 1.90 (1.50–2.15) | 1.82 ± 0.34; 1.90 (1.68–1.90) | 0.432 |
| IL-12 | Day 6 after exposure | 1.57 ± 0.39; 1.90 (1.20–1.90) | 1.60 ± 0.30; 1.55 (1.30–1.90) | 0.790 |
| IL-12 | Day 7 after exposure | 1.65 ± 0.31; 1.60 (1.45–1.90) | 1.46 ± 0.30; 1.40 (1.28–1.68) | 0.062 |
| IL-12 | Day 8 after exposure | 1.61 ± 0.24; 1.50 (1.45–1.90) | 1.48 ± 0.34; 1.35 (1.20–1.90) | 0.177 |
| IL-12 | Day 9 after exposure | 1.72 ± 0.54; 1.90 (1.30–1.90) | 1.59 ± 0.29; 1.50 (1.30–1.90) | 0.344 |
| TNF-α | Day 1 before exposure | 2.21 ± 0.74; 2.05 (1.78–2.50) | 2.84 ± 2.72; 2.10 (1.68–2.70) | 0.334 |
| TNF-α | Day 1 after exposure | 2.31 ± 0.85; 1.85 (1.68–3.03) | 2.57 ± 1.91; 1.85 (1.60–2.73) | 0.582 |
| TNF-α | Day 2 after exposure | 1.84 ± 0.75; 1.70 (1.35–1.90) | 2.95 ± 1.59; 3.20 (1.80–3.70) | 0.010 |
| TNF-α | Day 3 after exposure | 2.31 ± 0.93; 1.90 (1.50–3.10) | 1.89 ± 0.75; 1.65 (1.40–2.10) | 0.126 |
| TNF-α | Day 4 after exposure | 1.99 ± 0.56; 1.90 (1.55–2.25) | 2.26 ± 0.68; 2.00 (1.88–2.53) | 0.186 |
| TNF-α | Day 5 after exposure | 2.80 ± 0.91; 3.20 (1.95–3.70) | 2.68 ± 0.92; 2.45 (1.88–3.70) | 0.683 |
| TNF-α | Day 6 after exposure | 2.87 ± 0.99; 3.70 (1.85–3.70) | 3.05 ± 0.96; 3.70 (2.35–3.70) | 0.568 |
| TNF-α | Day 7 after exposure | 2.28 ± 1.17; 1.80 (1.65–2.25) | 1.96 ± 0.79; 1.65 (1.50–1.90) | 0.322 |
| TNF-α | Day 8 after exposure | 2.04 ± 0.81; 1.80 (1.50–2.15) | 1.81 ± 0.59; 1.70 (1.50–2.00) | 0.323 |
| TNF-α | Day 9 after exposure | 1.90 ± 0.74; 1.70 (1.50–1.95) | 2.01 ± 0.59; 1.90 (1.58–2.40) | 0.609 |

Values are mean ± SD; median (IQR). P values compare HOCl with NaCl at the corresponding time point in the source report. In Part B, the HOCl per-protocol population included 19 participants because one participant withdrew consent on Day 1 without providing a reason.

*One participant withdrew consent on Day 1 without providing a reason.

Abbreviations: HOCl, hypochlorous acid; IL, interleukin; NaCl, sodium chloride; TNF, tumor necrosis factor.

Supplementary Table 4. Local tolerability findings.

| Study part | Assessment | Outcome | HOCl group | NaCl group | P |
| --- | --- | --- | --- | --- | --- |
| **short repeated exposure cohort** | Laryngological examination | Baseline abnormality/NCS | 1/20 | 2/20 | 0.548 |
|  | Laryngological examination | Any post-exposure abnormality | 0/20 | 0/20 | 1 |
|  | Laryngological examination | Clinically significant finding | 0/20 | 0/20 | - |
|  | Conjunctiva | Baseline mild abnormality, left/right eye | 1/20 / 1/20 | 0/20 / 0/20 | 1 / 1 |
|  | Conjunctiva | Maximum post-exposure mild abnormality, left/right eye | 3/20 / 3/20 | 4/20 / 4/20 | 1/ 1 |
|  | Conjunctiva | Persistent abnormality at follow-up | 0/20 | 0/20 | - |
| **prolonged repeated exposure cohort** | Laryngological examination | Baseline abnormality/NCS | 0/20 | 4/20 | 0.11 |
|  | Laryngological examination | Clinically significant finding | 0/19 | 0/20 |  |
|  | Conjunctiva | Baseline mild/moderate abnormality, left eye | 7/20 mild; 1/20 moderate | 5/20 mild; 0 moderate | 0.64 |
|  | Conjunctiva | Baseline mild/moderate abnormality, right eye | 6/20 mild; 1/20 moderate | 4/20 mild; 0 moderate | 0.69 |
|  | Conjunctiva | Maximum post-exposure mild abnormality, left/right eye | 1/19 / 1/19 | 1/20 / 1/20 | 1 / 1 |
|  | Conjunctiva | Persistent abnormality at follow-up | 0/19 | 0/20 |  |

NCS, non-clinically significant.

Abbreviations: HOCl, hypochlorous acid; NaCl, sodium chloride; NCS, non-clinically significant.

Supplementary Table 5. Vital signs before and after exposure.

| Study part | Parameter | Time point | HOCl | NaCl | P |
| --- | --- | --- | --- | --- | --- |
| **short repeated exposure cohort** | Systolic BP, mmHg | Day 1 before exposure | 111.15 ± 14.65 | 110.65 ± 13.31 | 0.909 |
|  | Systolic BP, mmHg | Day 1 after exposure | 112.60 ± 11.58 | 113.90 ± 11.29 | 0.720 |
|  | Systolic BP, mmHg | Day 3 after exposure | 115.95 ± 11.28 | 111.10 ± 10.13 | 0.156 |
|  | Systolic BP, mmHg | Day 10 follow-up | 117.60 ± 10.66 | 116.90 ± 11.59 | 0.842 |
|  | Diastolic BP, mmHg | Day 1 before exposure | 79.45 ± 8.76 | 77.20 ± 7.55 | 0.391 |
|  | Diastolic BP, mmHg | Day 1 after exposure | 78.80 ± 6.55 | 78.80 ± 6.66 | 1.000 |
|  | Diastolic BP, mmHg | Day 3 after exposure | 78.30 ± 7.19 | 77.30 ± 7.17 | 0.663 |
|  | Diastolic BP, mmHg | Day 10 follow-up | 83.55 ± 6.30 | 81.05 ± 7.69 | 0.272 |
|  | Heart rate, bpm | Day 1 before exposure | 72.10 ± 9.92 | 69.95 ± 12.08 | 0.545 |
|  | Heart rate, bpm | Day 1 after exposure | 69.40 ± 10.30 | 63.45 ± 7.03 | 0.039 |
|  | Heart rate, bpm | Day 3 after exposure | 67.95 ± 9.54 | 66.55 ± 8.13 | 0.615 |
|  | Heart rate, bpm | Day 10 follow-up | 68.70 ± 7.57 | 68.75 ± 10.41 | 0.986 |
|  | Respiratory rate, breaths/min | Day 1 before exposure | 16.00 ± 1.47 | 16.60 ± 1.48 | 0.198 |
|  | Respiratory rate, breaths/min | Day 1 after exposure | 16.90 ± 1.01 | 17.15 ± 1.03 | 0.442 |
|  | Respiratory rate, breaths/min | Day 3 after exposure | 13.90 ± 1.10 | 14.10 ± 1.06 | 0.560 |
|  | Respiratory rate, breaths/min | Day 10 follow-up | 13.10 ± 0.30 | 13.20 ± 0.41 | 0.398 |
|  | Temperature, °C | Day 1 before exposure | 36.30 ± 0.19 | 36.30 ± 0.25 | 1.000 |
|  | Temperature, °C | Day 1 after exposure | 36.42 ± 0.15 | 36.30 ± 0.17 | 0.022 |
|  | Temperature, °C | Day 3 after exposure | 36.56 ± 0.25 | 36.55 ± 0.24 | 0.899 |
|  | Temperature, °C | Day 10 follow-up | 36.40 ± 0.29 | 36.45 ± 0.29 | 0.587 |

| **prolonged repeated exposure cohort** | Systolic BP, mmHg | Day 1 before exposure | 114.25 ± 15.00 | 118.35 ± 12.03 | 0.351 |
| --- | --- | --- | --- | --- | --- |
|  | Systolic BP, mmHg | Day 1 after exposure | 119.25 ± 15.17 | 117.95 ± 9.76 | 0.756 |
|  | Systolic BP, mmHg | Day 3 after exposure | 121.05 ± 11.78 | 122.50 ± 10.31 | 0.683 |
|  | Systolic BP, mmHg | Day 9 after exposure | 117.95 ± 14.84 | 118.65 ± 10.97 | 0.865 |
|  | Systolic BP, mmHg | Day 20 follow-up | 122.74 ± 12.48 | 123.30 ± 11.89 | 0.887 |
|  | Diastolic BP, mmHg | Day 1 before exposure | 76.00 ± 6.98 | 77.00 ± 7.96 | 0.680 |
|  | Diastolic BP, mmHg | Day 1 after exposure | 79.10 ± 5.93 | 79.00 ± 7.51 | 0.964 |
|  | Diastolic BP, mmHg | Day 3 after exposure | 82.21 ± 5.78 | 80.35 ± 4.66 | 0.280 |
|  | Diastolic BP, mmHg | Day 9 after exposure | 79.47 ± 7.44 | 78.95 ± 6.39 | 0.814 |
|  | Diastolic BP, mmHg | Day 20 follow-up | 81.58 ± 6.48 | 79.60 ± 6.83 | 0.359 |
|  | Heart rate, bpm | Day 1 before exposure | 75.25 ± 10.14 | 73.00 ± 10.69 | 0.503 |
|  | Heart rate, bpm | Day 1 after exposure | 67.60 ± 9.54 | 66.20 ± 10.06 | 0.660 |
|  | Heart rate, bpm | Day 3 after exposure | 67.05 ± 8.62 | 69.75 ± 11.51 | 0.412 |
|  | Heart rate, bpm | Day 9 after exposure | 69.26 ± 8.77 | 69.50 ± 11.39 | 0.942 |
|  | Heart rate, bpm | Day 20 follow-up | 76.74 ± 10.73 | 75.05 ± 9.38 | 0.603 |
|  | Respiratory rate, breaths/min | Day 1 before exposure | 15.35 ± 1.37 | 15.60 ± 1.08 | 0.542 |
|  | Respiratory rate, breaths/min | Day 1 after exposure | 14.75 ± 1.06 | 15.00 ± 1.15 | 0.478 |
|  | Respiratory rate, breaths/min | Day 3 after exposure | 14.74 ± 1.31 | 14.65 ± 1.58 | 0.846 |
|  | Respiratory rate, breaths/min | Day 9 after exposure | 13.73 ± 0.55 | 13.55 ± 0.50 | 0.285 |
|  | Respiratory rate, breaths/min | Day 20 follow-up | 14.05 ± 0.77 | 14.05 ± 0.81 | 1.000 |
|  | Temperature, °C | Day 1 before exposure | 36.52 ± 0.28 | 36.44 ± 0.31 | 0.402 |
|  | Temperature, °C | Day 1 after exposure | 36.40 ± 0.24 | 36.36 ± 0.21 | 0.587 |
|  | Temperature, °C | Day 3 after exposure | 36.40 ± 0.31 | 36.47 ± 0.39 | 0.541 |
|  | Temperature, °C | Day 9 after exposure | 36.40 ± 0.28 | 36.50 ± 0.33 | 0.306 |
|  | Temperature, °C | Day 20 follow-up | 36.50 ± 0.29 | 36.30 ± 0.26 | 0.029 |

Values are mean ± SD. The table includes baseline, first post-exposure measurement, final exposure day, and follow-up.

Abbreviations: BP, blood pressure; HOCl, hypochlorous acid; NaCl, sodium chloride.

Suppl. Fig. 1. Local tolerability findings by exposure regimen and treatment group. Heat maps showing the percentage of participants with abnormalities on laryngological and ocular assessments. Panels I and III show the short repeated exposure cohort (part A), and Panels II and IV show the prolonged repeated exposure cohort (part B). Panels I and II present laryngological findings, whereas Panels III and IV present ocular findings. Values indicate the percentage of participants with abnormalities at each scheduled assessment. CS, clinically significant; HOCl, hypochlorous acid; NaCl, sodium chloride; NCS, non-clinically significant.
